# Sodium alginate/carboxymethyl cellulose films embedded with liposomes encapsulated green tea extract: characterization, controlled release, application

**DOI:** 10.1039/d3ra05196j

**Published:** 2024-01-02

**Authors:** Xin Feng, Yang Li, Zhuoyu Cui, Rongrong Tang

**Affiliations:** a Department of Forestry Engineering, Northeast Forestry University Harbin Heilongjiang China liya@nefu.edu.cn; b Department of Logistics Engineering and Management, Northeast Forestry University Harbin Heilongjiang China

## Abstract

To maintain the freshness of the fruit during storage, sodium alginate/carboxymethyl cellulose films embedded with pH-senstive liposomes encapsulated green tea extract were developed (SA/CMC/TP-Lip). An orthogonal design was used to optimise the preparation of TP-Lip and SA/CMC/TP-Lip was prepared through response surface. The stability of TP-Lip structure was measured. The morphology of SA/CMC/TP-Lip was characterised by SEM, and the mechanical properties and oxidation resistance of films were measured. Special attention was paid to the pH sensitivity of TP-Lip and the improvement of film properties. The zeta potential and encapsulation rate of TP-Lip were −45.85 ± 2.13 mV and 61.45 ± 0.23%. The average release rate of TP encapsulated into TP-Lip at pH 3 was 41.08%, an increase of 23.07% over pH 6 during 12 h. SEM and FTIR showed that TP-Lip was structurally stable and had good compatibility with SA/CMC. Tensile strength was increased by 30.55% and DPPH radical scavenging capacity was increased by 7.16% with the addition of TP-Lip. SA/CMC/TP-Lip is applied to blueberries to reduce their weight loss and improve the loss of freshness of blueberries during storage. Thus, SA/CMC/TP-Lip could provide a new way to extend active packaging materials and maintain fruit freshness during storage.

## Abstract 2

To maintain the freshness of the fruit during storage, sodium alginate/carboxymethyl cellulose films embedded with pH-senstive liposomes encapsulated green tea extract were developed (SA/CMC/TP-Lip).

## Graphical abstract

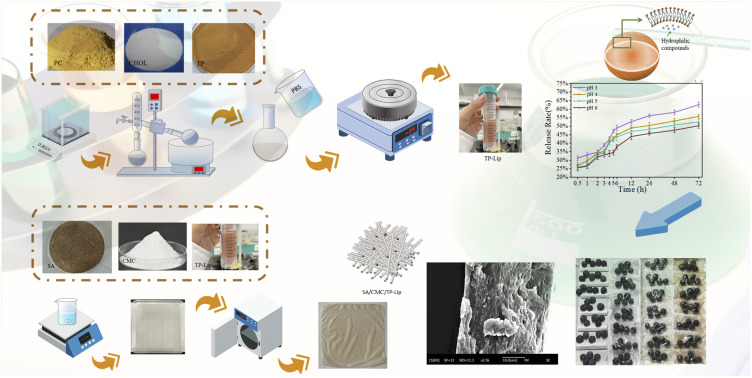

## Introduction

Fruits are highly prone to deterioration during storage and transportation, necessitating the implementation of methods to prolong their shelf life. Blueberries, recognized as the “Queen of Fruits” and listed among 5 “Healthy Fruits for Humanity” by the FAO, possess cancer-fighting, prevent cardiovascular disease-preventing, and neurological decline-preventing properties.^1–5^ Consequently, the development of packaging materials capable of preserving the freshness of blueberries during storage.^6,7^

The detrimental effects of synthetic and petroleum-based plastic materials on the marine environment, human health, and species diversity are well documented. However, due to the continued high demand for materials in society, there is a growing interest in environmentally friendly alternatives such as biopolymers composed of proteins, polysaccharides, and lipids.^8–10^ Among these alternatives, sodium alginate (SA) and carboxymethylcellulose (CMC) have gained significant popularity.^11–14^ SA, derived from brown algae, is a water-soluble and non-toxic natural polysaccharide. It contains a substantial amount of –COO ^–^ groups and exhibits polyatomic behavior in aqueous solutions, displaying a notable sensitivity to changes in pH.^15–18^ SA is a “Generally Recognised as Safe” (GRAS) raw material, often used as a stabiliser and thickener in the food industry.^19^ CMC is a negatively charged cellulose derivative derived from natural cellulose.^20–22^ It is soluble in water, has excellent biocompatibility and good viscosity, is stable to light and heat, is non-toxic and non-allergenic, and is ordinarily prepared as a substrate for active packaging.^23–26^ Meanwhile, SA and CMC can produce strong intermolecular interactions between the polymers through hydrogen bonding, which improves the mechanical properties of the film, resulting in a film with better performance and lower cost.^27^ Nonetheless, there has been no detailed research on the combination of SA, CMC, and other materials into new active packaging, and the effect of adding different materials on the packaging is unknown.

Tea polyphenols (TP) extracted from the leaves of the evergreen tea tree (*Camellia sinensis* L.) are the main chemical constituents of tea with health benefits and are commonly used in the food industry for their antibacterial and antioxidant abilities.^28–31^ TP is the most popular natural antioxidant and is widely combined with some natural biopolymers (polysaccharides and proteins) into active packages for food applications. However, TP is heat resistant and stable under acidic conditions, it is susceptible to oxidative polymerization under light, and these active packages usually do not achieve satisfactory results during storage.^32–34^ Therefore, many studies have started to focus on improving the stability of TP in reactive packaging.

The use of natural antioxidants in films may be affected by the external environment (pH, light, and heat), resulting in unstable properties. Therefore, the use of micro-nano-encapsulation and various nanocarriers (nanoliposomes) in active packaging has increased.^35–38^ The structure of nanoliposomes can improve the stability of antioxidants, effectively control the release rate of TP components, and prolong the service life of active packaging.^39^ Nonetheless, the loading of liposomes on the film will change the properties. Whether this novel packaging can be useful in food preservation is also a focus of future research, and the study will provide favorable assistance for the development of new active packaging.

This prospective study was designed to develop a novel film with controlled release and oxidation resistance, to analyse the release behaviour of liposomes at acidic pH, to measure various properties of liposomes and films, and finally to apply films in low temperature fruit preservation. The film can control the release of antioxidants and provide a new research design and method for active packaging.

## Experimental

### Materials

SA, glycerol, Tween-80, potassium dihydrogen phosphate and disodium hydrogen phosphate were provided by Sinopharm Chemical Reagent Co. (Jingan, Shanghai, China). CMC with a nominal viscosity of 1200–1600 was provided by Tianjin Comio Chemical Reagent Co. (Jinnan, Tianjin, China). Lecithin High Potency (PC), green tea extract (TP ≥ 98.5%), and cholesterol (CHOL) were provided by Shanghai Shifeng Biotechnology Co., Ltd. (Baoshan, Shanghai, China). 1,1-Diphenyl-2-picrylhydrazyl (DPPH) was provided by Fuzhou Feijing Technology Co. (Minhou, Fuzhou, China).

### Preparation of TP-Lip

TP liposomes (TP-Lip) were prepared using the method of Cui^40^ with some modifications. Appropriate amounts of PC, CHOL, and 0.2 mL of Tween-80 were dissolved in 20 mL of anhydrous ethanol containing appropriate amounts of TP. The organic solvent was removed from the mixture using a rotary evaporator (RE-52CS-2, Shanghai Yarong Biochemical Instrument Factory, Minhang, Shanghai, China) at 40 °C to form a homogeneous film. The film was hydrated with 30 mL of 0.1 M phosphate-buffered saline (PBS) at pH 7.2 for 90 min (20 °C). Afterward, the solution was filtered through a 0.22 μm membrane and stored at 4 °C until used.

### TP content measurement

To estimate the TP content, a modified method was used.^41^ The UV absorption spectra of TP were measured using a UV spectrophotometer (T6, Beijing General Analytical Instruments Co., Ltd., Pinggu, Beijing, China) with a 200–700 nm scanning region. The TP samples were weighed to prepare standard solutions of 30, 60, 120, 180, 240, and 300 μg mL^−1^. The absorbance of each standard solution was measured at 275 nm using anhydrous ethanol as the control group. The linear regression relationship between absorbance and concentration was calculated, and the regression equation obtained was: *y* = 0.108*C* + 0.004, *R*^2^ = 0.993.

### Encapsulation rate (EE) measurement

The EE of liposomes was measured using the method of Lu^42^ with slight modification. 400 μL of liposome solution was aspirated into a centrifuge tube, mixed with distilled water to 2 mL, and centrifuged at 12 000 rpm for 30 min at room temperature in a high-speed desktop centrifuge (TGL-20B, Shanghai Anting Scientific Instruments Factory, Jiading, Shanghai, China). Aspirate the supernatant and measure the absorbance at 275 nm. The results were entered into the following equation to calculate EE:
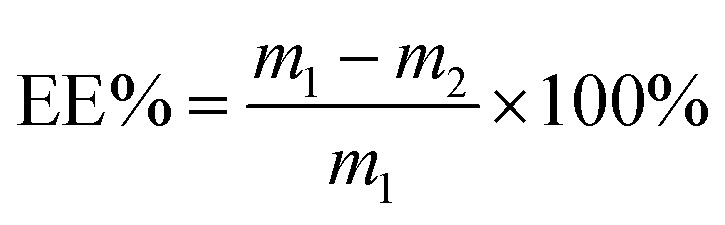
where *m*_1_ is the total amount of TP and *m*_2_ is the amount of TP in the solution.

### Single-factor and orthogonal experiments

Based on previous single-factor optimization experiments, the ratio of CHOL to PC, the ratio of TP to PC and CHOL, and the hydration temperature were used to select the orthogonal design level. The EE was used as an optimization index.

### Characterization of TP-Lip

#### Particle size, and zeta potential

The particle size and zeta potential of TP-Lip were determined using a laser particle sizer (Zeta Plus, Brookhaven Instruments, Holtsville, NY, USA).

#### Metallurgical microscopy

A volume of 200 μL of liposome solution was added dropwise to the slide, followed by 200 μL of anhydrous ethanol, dried naturally, and observed at 400× and 1000× magnification, respectively.

#### Release rate at different pH

A volume of 5 mL of liposome solution was stored in dialysis bags (MW8000-14000) in PBS of pH 3, 4, 5, and 6 and incubated in a thermostatic shaker (HZQ-C, Spectral Calibration Dongguan Laboratory Technology Co., Ltd., Dongwan, Guangdong, China.) at 25 °C with shaking at 100 rpm,^43,44^ and 0.5, 1, 2, 3, and 4, 5, 6, 12, 24, 48, and 72 h were sampled, and each sample was supplemented with an equal amount of PBS.

#### Antioxidant activity

The ability of blank liposomes and TP-Lip to scavenge DPPH was measured by the method of Caddeo.^45^ A 0.1 M DPPH standard solution was prepared. The UV absorption spectra were scanned at 340–700 nm using a UV spectrophotometer.^46^ Specifically, 2 mL of liposomes and 2 mL of anhydrous ethanol were added to 2 mL of DPPH standard solution, respectively, and the absorbance values at 516 nm were measured after standing for 40 min away from light.

#### Stability

The EE of liposomes was measured under different temperature treatments to study the physical stability of liposome samples using the method of^47^ with slight modifications, and samples were measured and analyzed after being placed at freeze-thaw (FT), 4 °C, and 20 °C for 2 weeks. The freeze-thaw (FT) group was stored at −20 °C and then thawed at room temperature for 4 h. The samples in the 4 °C and 20 °C groups were measured for EE after storage at 4 °C and 20 °C.

### Preparation of SA/CMC/TP-Lip

To prepare the film through drop casting,^48^ a certain amount of TP and TP-Lip was added to appropriate amounts of SA and CMC, 20 wt% of the total mass of the film solution was added to glycerol, and the mixture was stirred at room temperature until the solution was completely mixed and then dried at 40 °C for 24 h. The resulting dried films were equilibrated at 20 °C and 50% relative humidity (RH) for 48 h before being peeled off for further analysis.

### Response surface

The effects of three factors on film WVP were designed separately: the amount of SA added, the amount of CMC added, and the amount of TP-Lip added. The results of these single-factor experiments were used to derive the optimal preparation method using Box–Behnken Design (BBD) with WVP as the optimization objective.

### Characterization of SA/CMC/TP-Lip

#### Determination of mechanical properties

The samples were cut into strips (10 mm × 80 mm) using the method of^49^ with slight modifications. A computerized measurement and control tensile testing machine (LD-05, Changchun Yueming Small Experimentation Machine Sales Department, Changchun, China) with an initial distance of 30 mm and a measurement rate of 1 mm s^−1^.

#### Water vapor permeability (WVP)

The WVP of SA/CMC/Lip films was measured using the method of^50^ with slight modifications. Film samples were cut into circles and covered in glass vials containing 2.0 g CaCl_2_ (0% RH), which were placed at room temperature and then exposed to a desiccator containing a saturated NaCl solution (75% RH), and the weight of the vials was measured after 24 h.
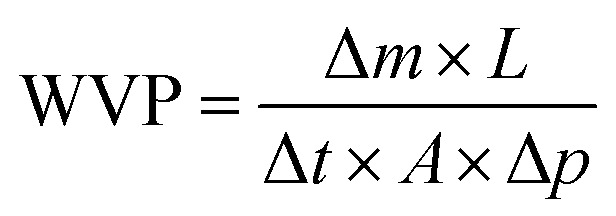
where Δ*m* (g) represents the weight increase of the glass bottle, *L* (mm) represents the average thickness of the film, Δ*t* (h) represents the glass bottle placement time, *A* (cm^2^) represents the permeation area of the film sample, and Δ*p* (Pa) represents the vapor pressure.

#### Fourier transform infrared spectroscopy (FTIR)

The infrared spectra of the films were measured using the method of Zhou with minor modifications,^48^ and the samples were scanned at 4000 to 500 cm^−1^ using a Fourier transform infrared spectrometer (Frontier, PerkinElmer Co., Ltd., Waltham, MA, USA) with the resolution chosen for the scan 2 cm^−1^.

#### SEM

SEM (EM-30 Plus, COXEM Co., Ltd., Yuseong-gu, Daejeon, Korea) was used to observe the surface and cross-sectional morphology of the films. The films were cut into small pieces (10 mm × 5 mm), followed by gold spraying of the samples, and then imaged at an operating voltage of 15 kV.

#### Antioxidant activity of films

The samples (30 mm × 30 mm) were mixed with distilled water in a bottle and incubated with gentle shaking at 20 °C. After 24 h of shaking, the DPPH (516 nm) radical scavenging rate was calculated using the formula.

#### Application

Fresh undamaged blueberries were wrapped in ordinary cling film, SA/CMC film, and SA/CMC/Lip film and refrigerated at 4 °C. Blueberries without packaging were sampled at 2d intervals and the quality data including weight loss, hardness, pH, and total soluble solids content (TSS) were compared between packages on the same sampling day.

### Statistical analysis

Orthogonal and response surface experiments were designed and analyzed by Design-Expert 13, and the experimental data were organized, edited and analyzed using SPSS 26, *p* < 0.05. Graphic drawing was performed using Origin 2021.

## Results and discussion

### Optimized preparation of TP-Lip

**Fig. 1:**
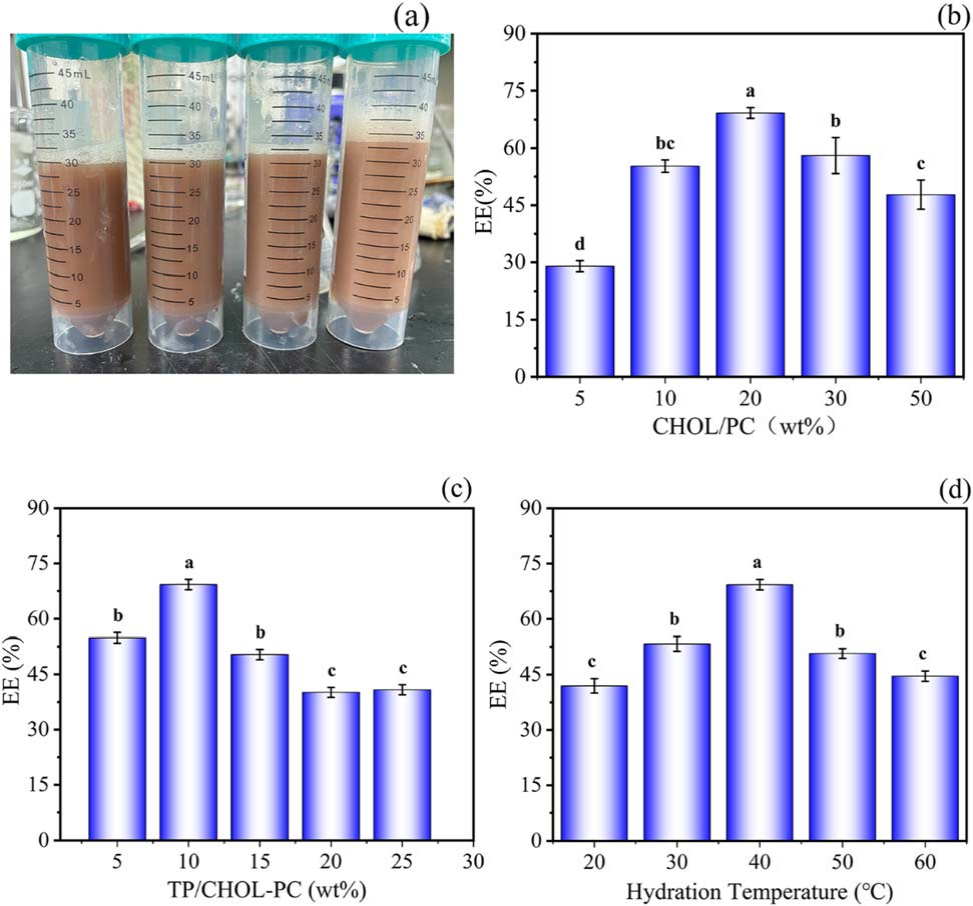
Single-factor experiments of liposomes. Liposome suspension (a), effect of CHOL/PC on EE (b), effect of TP/CHOL-PC on EE (c), effect of hydration temperature on EE (d).

Through reading the papers and previous experiments, it was found that CHOL/PC, TP/CHPL-PC, and hydration temperature had the greatest effect on the EE of TP-Lip,^51^ thus these three factors were selected for optimization experiments. The single-factor experiment was performed to exclude the levels that had little effect on the EE, and then on the basis of the results of the single-factor experiment, the following experimental levels were selected: CHOL/PC (10 wt%, 20 wt%, 30 wt%), TP/CHOL-PC (5 wt%, 10 wt%, 15 wt%), and hydration temperature (30 °C, 40 °C, 50 °C) (Table 1). The color of the prepared TP-Lip solution was reddish brown, as shown in (Fig. 1a). The best fabrication methods were CHOL/PC with 30 wt%, TP/CHOL-PC with 5 wt%, and hydration temperature of 40 °C.

**Table 1:** Orthogonal analysis table (*p* < 0.05)

RUN	CHOL/PC (wt%)	TP/CHOL-PC (wt%)	Hydration temperature (°C)	EE (%)
1	30	10	30	62.23
2	30	5	50	67.38
3	20	10	50	50.72
4	30	15	40	65.58
5	20	5	40	54.87
6	10	10	40	55.28
7	10	5	30	58.37
8	20	15	30	55.02
9	10	15	50	54.10

Cholesterol affected the mobility of liposomes by acting as a stabilizer and reducing the leakage of compounds included in liposomes. As cholesterol and the drug compete for the hydrophobic region of the lipid bilayer, EE can be reduced by too much cholesterol.^52^ Furthermore, when the amount of lecithin in liposomes was too low (Fig. 1b), liposome membranes were not easily formed, and a low lecithin to cholesterol mass ratio made the phospholipid bilayer not tightly arranged,^53^ which was responsible for the decrease in EE.

The TP content played a significant role in the encapsulation efficiency (EE) of liposomes. A lower TP content led to a decrease in EE as less TP was encapsulated by the liposomes. Conversely, a higher TP content exceeded the maximum limit of lipid encapsulation, resulting in an increase of free TP content in the solution and subsequently decreasing the EE (Fig. 1c). These align with previous studies.^54^

Additionally, the relatively high EE observed in liposomes hydrated at temperatures between 30 and 50 °C can be attributed to the phase transition temperature of soy lecithin within this range (Fig. 1d) as stated in,^55^ the phospholipid bilayer underwent a transition from the gel phase to the liquid crystal phase at a temperature of 34.39 °C. Consequently, the hydration temperature exceeded the phase transition temperature, leading to improved liposome formation. However, excessively high temperatures can accelerate the oxidation of soy lecithin.^56^

### Characterization of TP-Lip

**Fig. 2:**
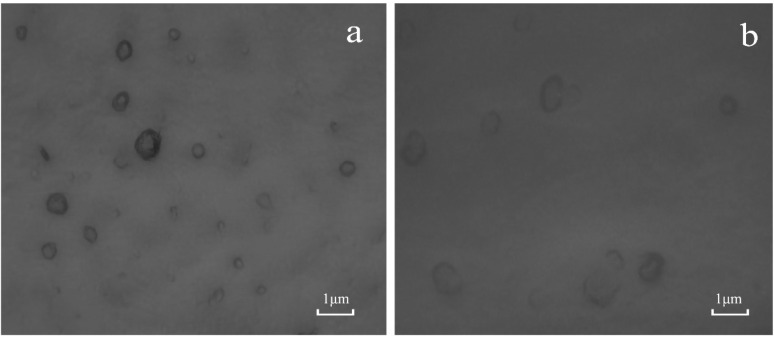
Microstructure of liposomes. 400× under a microscope (a) and 1000× under a microscope (b).

The morphology of TP-Lip was depicted in (Fig. 2), exhibiting an ellipsoidal shape, and the liposomes incorporating TP exhibited an intact vesicle structure,^57^ indicating a relatively successful liposome encapsulation. The TP-Lip system exhibited an average particle size of 810.48 ± 1.83 nm, a zeta potential of −45.85 ± 2.13 mV, and an EE of 61.45 ± 0.23. These findings indicate that the liposome formulation displayed a relatively stable nature. However, it is worth noting that the liposomes had a slightly larger average particle size and a tendency to aggregate into flocs due to the presence of TP.

### Stability of TP-Lip

To assess the physical stability of TP-Lip, the EE was measured at different storage temperatures. Notably, significant differences in the EE of TP-Lip were observed over the course of the storage period. The TP-Lip remained relatively stable during storage at 4 °C, which aligns with previous research.^58^ However, the changes in TP-Lip became more noticeable after 2 weeks of storage at both freezing-thawing (FT) and 20 °C, with the highest average loss in encapsulation efficiency recorded as 7.61% after 20 °C storage (Table 2). TP-Lip exhibited significant instability under FT conditions, which can be attributed to the disruption of liposome structure caused by the freeze-thaw process. The mechanical stress and the increase in solute substances during FT further aggravated the chemical disruption of the structure, resulting in the leakage of active substances and a decrease in EE.^47^ Additionally, as mentioned in,^59^ the hydrophobic lumen of the liposome membrane surface and the membrane permeability also underwent changes during storage, contributing to the decrease in liposome stability after freeze-thawing.

**Table 2:** EE of 4 °C, 20 °C, FT group. (*p* < 0.05)

EE (%)	4 °C	20 °C	FT
2 weeks	61.23 ± 1.06^a^	57.40 ± 0.56^b,c^	58.67 ± 0.78^b^

### Release rate at different pH

**Fig. 3:**
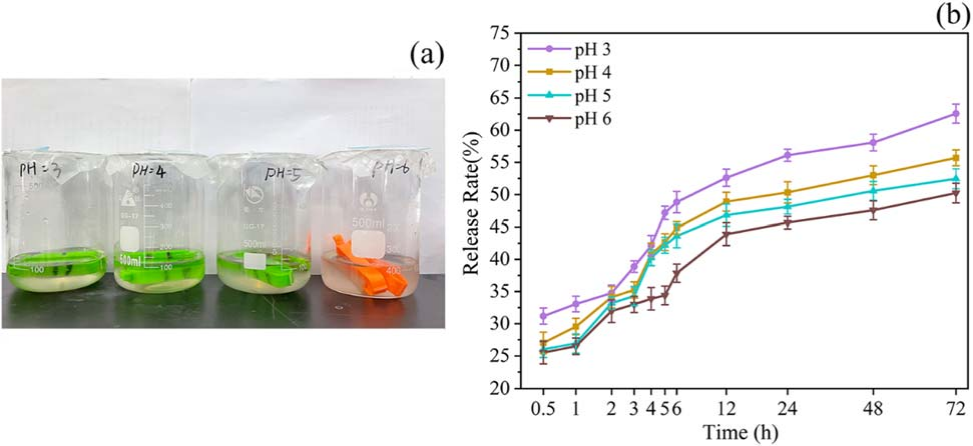
Effects of different pH on the release rate. Samples placed at different pH (a), release rates at different pH (b).

At pH levels of 3, 4, 5, and 6, the release rate of TP increased continuously. The increase was particularly dramatic in the first 12 h and gradually leveled off after 24 h (Fig. 3). This phenomenon could be attributed to the sensitivity of PE in phospholipids to acidic conditions, causing internal structural changes. These changes resulted in the breakage of the molecular skeleton and the subsequent release of the active substances contained within. On the other hand, the low pH levels could trigger hydrolysis reactions in phospholipid bilayers, further contributing to the increased release rate of TP.^60^ Following the initial release burst period, the remaining TP in the liposome was slowly released over time, allowing for controlled release functionality.

### Antioxidant activity

TP was a good antioxidant, that could capture free radicals through phenolic hydrogen, scavenge free radicals, and block the chain reaction of free radicals to achieve the antioxidant effect. 40.76 ± 0.57% of free radicals were scavenged by TP-Lip, which had a good antioxidant capacity and was close to the antioxidant capacity reported by,^55^ further demonstrating that TP was effectively encapsulated and could function properly.

### Optimized preparation of SA/CMC/TP-Lip

**Fig. 4:**
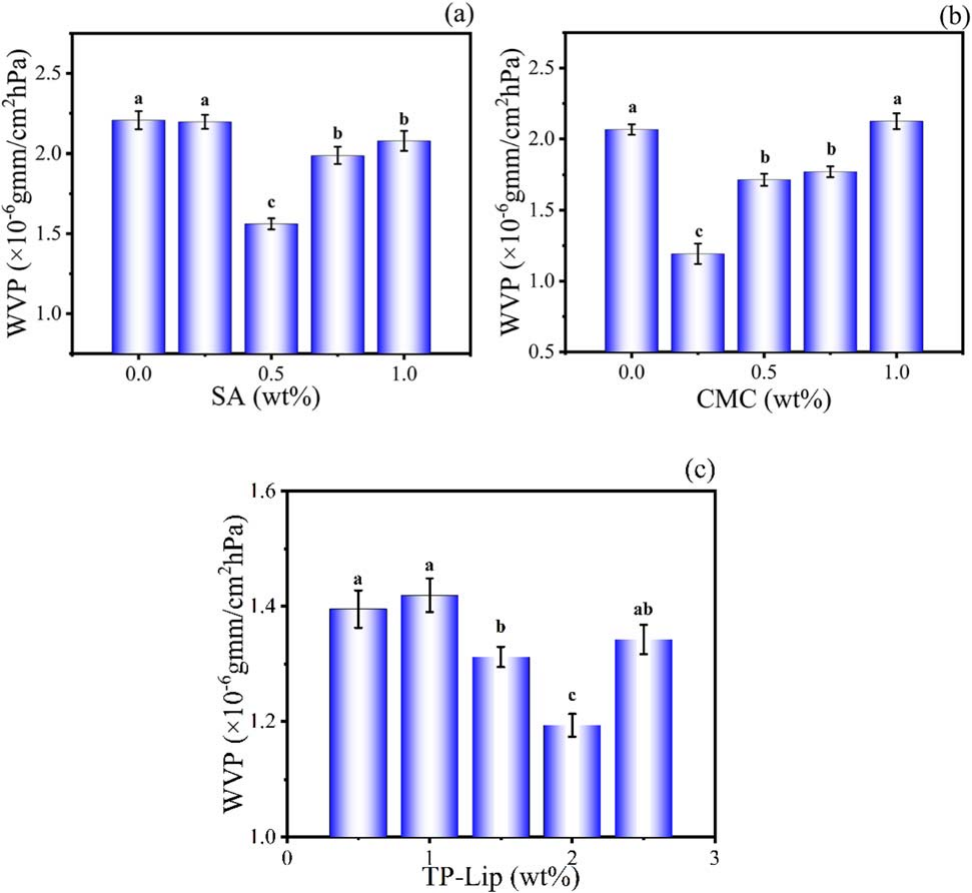
Single-factor experiments of the film. Effects of SA addition on film WVP (a), CMC addition on film WVP (b), and TP-Lip addition on film WVP (c).

The high WVP was a significant issue with polymer films, as it could affect the diffusivity and solubility of water molecules within the films. The infiltration of moisture into the polymer network structure could affect the quality and shelf life of the compound. Therefore, reducing WVP could extend the lifespan of the packaging material and minimize changes in the internal environment. The hydrophilic groups (hydroxyl and carbonyl) of SA form hydrogen bonded with CMC in the film, reducing the number of active sites exposed on the surface of the film, thus the interaction between hydrophilic groups and moisture possibilities was limited.^61^ The weak barrier properties of CMC to water depended on the presence of hydrophilic groups in the polymer matrix.^62^ In this study, the range of values optimized by a single-factor experiment was selected for SA addition (0.5 wt%, 0.75 wt%, 1 wt%), CMC addition (0.25 wt%, 0.5 wt%, 0.75 wt%), and Lip addition (1.5 wt%, 2 wt%, 2.5 wt%) for BBD design (Fig. 4).^63^ During fruit storage, the gas concentration in the room changed due to respiration, and the pH in the room is affected by water vapour, thus the film was optimised for the water vapour transmission rate. As the number of liposomes increased, the internal molecular arrangement of the film with different crystal arrangements was unbalanced, resulting in cracks on the surface and increasing the WVP. The optimal preparation process consisted of using 0.791 wt% SA, 0.326 wt% CMC, and 1.856 wt% TP-Lip.^64^

**Fig. 5:**
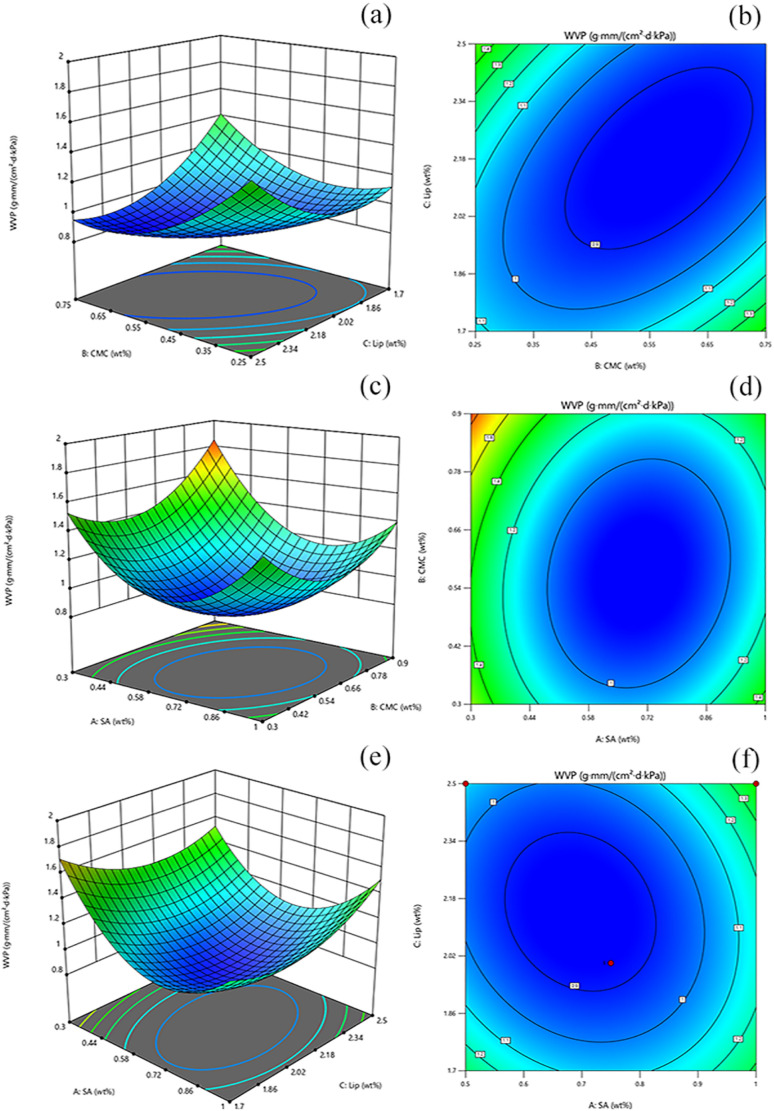
Response surface optimization of the film. Response surface (a) and contours (b) for CMC/TP-Lip to WVP, response surface (c) and contours (d) for SA/CMC to WVP, and response surface (e) and contours (f) for SA/TP-Lip to WVP.

The effect of interactions between the factors could be assessed by examining 3D response surface plots and two-dimensional contour plots.^65^ The response surface plots and contour plots of the interaction of each experimental factor were shown in (Fig. 5). The plots for the interaction of CMC and TP-Lip, CMC and SA, and SA and TP-Lip all opened upward with steep slopes, and their contour plots tended to be more elliptical. This suggests that all of these interaction terms had a significant effect on WVP (*p* < 0.05). The ANOVA results confirmed the significance of the model with *p* < 0.05, and the fitted correlation coefficient *R*^2^ was 0.9572, indicating that the model was significant. The model developed based on the coding factors was as follows:WVP ≡ 0.8710 + 0.0690 × *A* − 0.0049 × *B* − 0.1592 × *C* − 0.0564 × *AB* + 0.0864 × *AC* − 0.2575 × *BC* + 0.2235 × *A*^2^ + 0.1898 × *B*^2^ + 0.3096 × *C*^2^where *A* is the amount of SA added, *B* was the amount of CMC added, and *C* was the amount of Lip added.

### Performance

To maintain the structural integrity of the packaging material, tensile strength (TS) and elongation at break (EAB) were considered. The EAB of SA/CMC film was 30.29 ± 0.24% and TS was 24.45 ± 0.12 MPa. The higher EAB value of SA/CMC was attributed to the presence of a large number of free hydroxyl groups on the surface of SA/CMC film, which increased molecular mobility.^66^ When Lip was added the TS of the SA/CMC membrane was greatly improved to 31.87 ± 0.25 MPa, while also maintaining a good EAB of 24.08 ± 0.24%. This finding was consistent with the results of Hashemi *et al.*^67^ and Didar *et al.*^68^ The incorporation of oregano and thyme nanoemulsions into methylcellulose-based films by Hashemi led to an increase in the TS of the films, a phenomenon that could be attributed to the network enhancement that occurred in the presence of large hydrogen bonds (dipole–dipole interactions) between the polar groups, especially hydroxyls in the polymer structure, and the polar headedness of the surfactant molecules at the dispersed interface of the nanodroplets. Ghasempur^69^*et al.* pointed out that there was a direct correlation between the TS of the films and the content of the nanoliposomes and attributed it to the presence of lecithin in the nanoliposome structure.

### WVP

Waterproof performance was of utmost importance in food packaging, particularly when it came to evaluating the moisture-proof ability of films used in food packaging, especially for fruits and vegetables.^18^ A lower WVP could reduce the moisture loss of packaged foods to the atmosphere, which was a significant factor in postharvest vegetable spoilage.^70^ The WVP of the film prepared according to the optimal preparation process was 1.09 × 10^−10^ g m^−1^ s^−1^ pa^−1^. In a study conducted by Abdin *et al.*,^64^ they were able to decrease the WVP of the film by incorporating thymus purified extract into CMC and SA membranes (1.121 × 10^−10^ g m^−1^ s^−1^ pa^−1^). This suggested that the addition of appropriate TP-Lip to CMC and SA could reduce the WVP of the film. In another study by Wang *et al.*^71^, the addition of cinnamon essential oil to chitosan films changed the single structure of chitosan and made it denser. At the same time, the hydrophobicity of cinnamon essential oil prevented the diffusion of water vapor through the components and reduced the WVP of the film.

### Morphology and structure

**Fig. 6:**
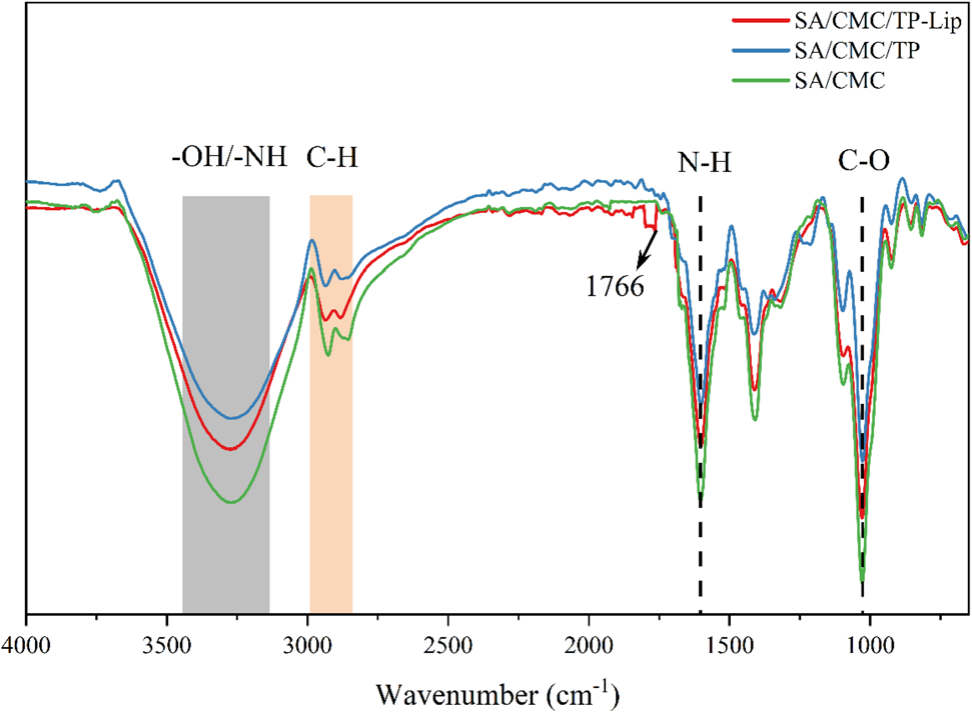
FTIR spectra of the film.

FTIR spectra of the three films were compared to determine the chemically reactive functional groups and their interactions in the polymer matrix (Fig. 6). The characteristic peaks associated with SA and CMC were detected,^66^ these peaks included –OH and –NH bonds (3200–3500 cm^−1^) stretching vibrations, stretching vibrations of C–H bonds (nearly 3000 cm^−1^), stretching vibrations of N–H at 1606 cm^−1^ and C–O bonds at 1028 cm^−1^. Additionally, SA/CMC/Lip films showed an absorption peak of C=O at 1766 cm^−1^ and the occurrence of this phenomenon was a stretching vibration of the ester C=O group^1^ of phosphatidylcholine.^72^

**Fig. 7:**
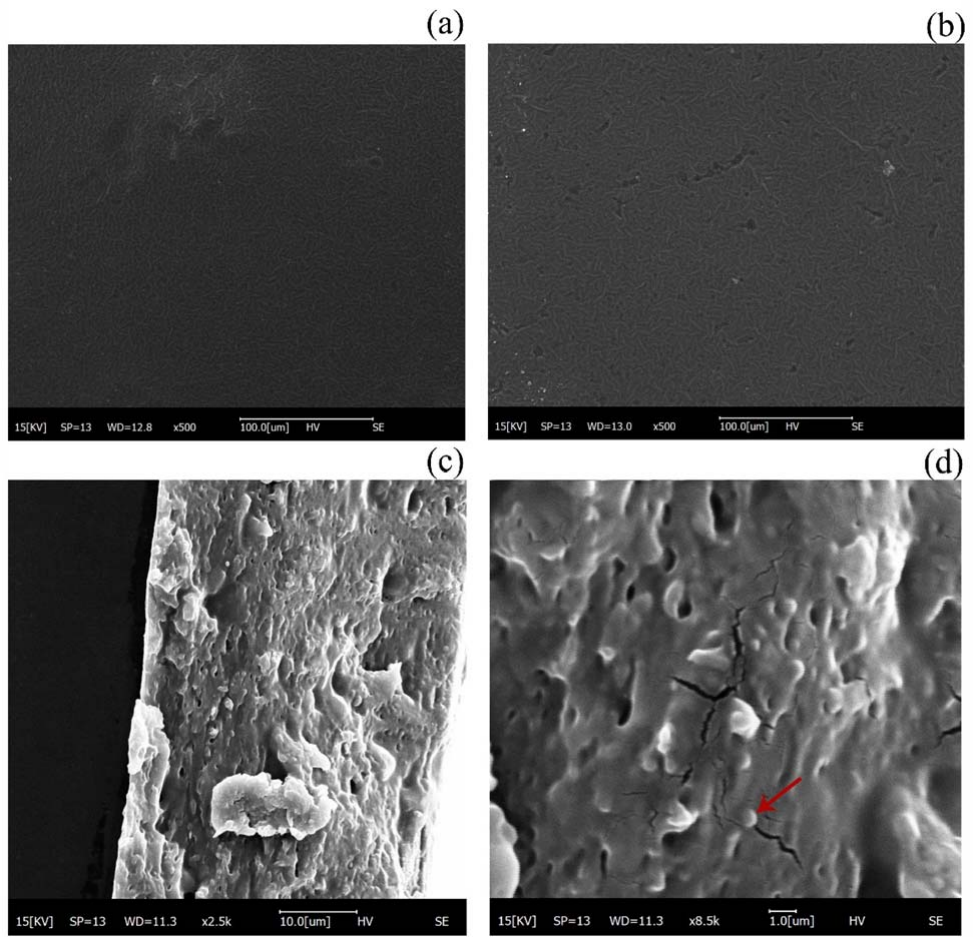
Microstructure of the film. SEM of SA/CMC membrane (a), scanning electron micrographs of SA/CMC/TP-Lip membrane (b)–(d).

Microstructure and morphology played a crucial role in the study of biofilms, which affected various properties of the film samples and could visualize changes in the internal structure of the films, indirectly demonstrating the effect of material compatibility. The structure and surface formation of SA/CMC and SA/CMC/TP-Lip film matrices were investigated through SEM analysis (Fig. 7). All prepared samples showed dense morphological surface structures, which confirmed the good miscibility between the materials used for film formation. However, some depressions were seen on the surface of the SA/CMC/TP-Lip film, which were due to the addition of TP-Lip, causing folds on the surface of the film. In cross-sectional Fig. 7c and d of SA/CMC/TP-Lip, numerous vesicle-like structures could be observed^48^ with smooth surfaces, and the diameters of these structures were roughly between 800–900 nm, showing the successful incorporation of TP-Lip into the SA/CMC films.

### Antioxidant activity and application

Antioxidant activity played a crucial role in the preservation of foods, particularly high-fat foods. The DPPH radical scavenging assay was a method used to evaluate antioxidant activity. The mechanism of this method involved the conversion of DPPH (2,2-diphenyl-1-picrylhydrazyl) free radicals into yellow molecules (2,5-diphenyl-1-picrylhydrazyl) by antioxidants. The extent of this reaction was determined by the hydrogen-donating capability of antioxidants.^73,74^ The ability to scavenge DPPH radicals effectively delayed oxidative deterioration and prolonged the shelf life of products.^35^ The film without TP-Lip addition exhibited a DPPH radical scavenging rate of 42.58 ± 1.56%, whereas the active film with TP-Lip addition showed a scavenging rate of 45.63 ± 2.03%, indicating a 7.16% improvement. This highlighted the remarkable antioxidant capacity of the SA/CMC/TP-Lip film.

The spoilage of blueberries was commonly caused by physiological and microbial processes, as well as their interactions. Blueberries were particularly susceptible to the production of some of the bacteria or fungi responsible for their spoilage after harvest, such as *Escherichia coli*, *Staphylococcus aureus*, and *Streptococcus*. In addition, microorganisms could utilize fermentable carbohydrates in food products to produce metabolites that result in the deterioration of the sensory properties of the product, such as pectinases, cellulases, and proteases.^75^ The blueberries were often exposed to various forms of oxidative damage, which was typically initiated by lipoxygenase (LOX) or exposure to factors such as heat, ionizing radiation, metal ions, light, and metalloprotein catalysts.^76^ This oxidation process could lead to the production of reactive oxygen species (ROS), which in turn could result in the deterioration of the nutritional quality, color, texture, taste, and flavor of the food. Ultimately, this reduces the overall shelf life of the food.^77^

**Fig. 8:**
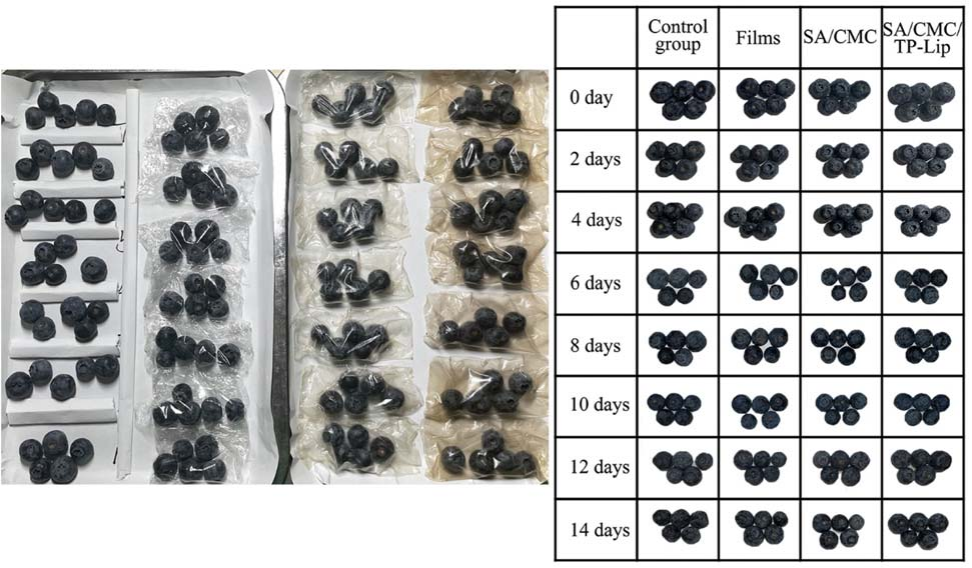
Blueberries during storage.

**Fig. 9:**
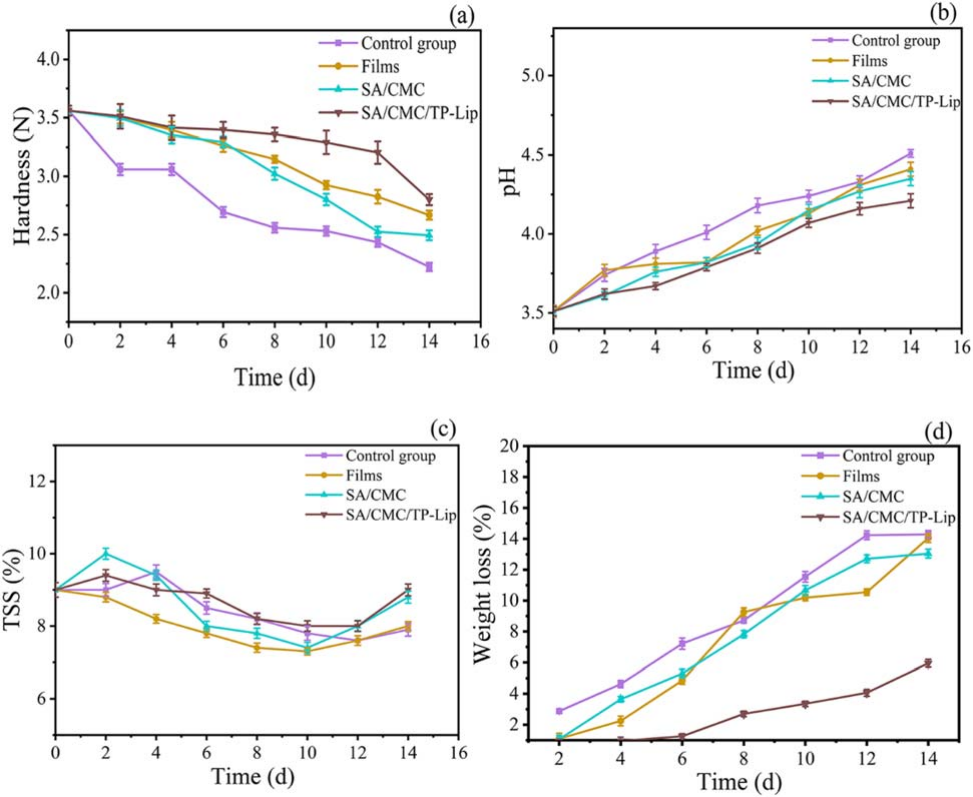
Changes in physicochemical indexes of blueberries. Changes in hardness (a), pH (b), TSS (c), and weight loss (d) during storage of blueberries.

Hardness was an important indicator that influenced consumers to purchase fruits and could be observed to determine the freshness of the fruit. During storage, blueberries tended to dehydrate, resulting in skin wrinkling (Fig. 8). Additionally, they were prone to harboring microorganisms and bacteria, leading to a shorter shelf life of 3–5 days in the refrigerator. The hardness of blueberries generally decreased over time. Nonetheless, the hardness of the SA/CMC/TP-Lip group decreased slowly and was 25.97% higher than the control group after 2 weeks of storage (Fig. 9a), showing that the new active packaging could effectively reduce the degradation of cell wall material and reduce fruit softening. The higher decrease in hardness in the SA/CMC/TP-Lip group at the later stages of hardness was due to the long storage period, which changed the water content in the fruit and affected the activity of enzymes in the fruit tissues, water loss increased the pectinase and polygalacturonase activity, resulting in fruit softening.^8^

The changes in pH and TSS content of the samples during storage are shown in Fig. 9b and c, respectively. The pH of all samples was increasing, which was related to the gradual metabolism and reactions of the samples. Blueberries underwent metabolic and chemical reactions during storage, converting starch and acid into sugar, resulting in an increase in both indexes.^78^ Furthermore, when amino acids were broken down, the fruit accumulated ammonia and amines, which triggered an increase in pH. The TSS content showed an overall increasing trend followed by a decrease. The initial metabolism of fruits converts carbohydrates into sugars and other soluble compounds, and the fruit starts to accumulate TTS, followed by a decrease in TTS as respiration requires nutrients.^79^ In addition, the longer the storage period, the more damage occurred to the cell structure, accelerating the loss of TSS.

Weight loss during storage was associated with fruit respiration and transpiration, which would lead to water loss and weight loss. The weight loss rate was also affected by atmospheric conditions, temperature, and water pressure gradients between fruit tissues. Over the storage period, the weight loss rate of each group of blueberries gradually increased (Fig. 9d). Among the different groups, SA/CMC/TP-Lip exhibited a relatively stable weight loss rate from 0 to 4 days but started to increase thereafter. It showed that SA/CMC/TP-Lip effectively reduces water evaporation, decreases the weight loss rate of blueberries, and extends their freshness period.^80^

## Conclusions

In this study, the optimal preparation process for TP-Lip and SA/CMC/TP-Lip was determined. The good performance of TP-Lip was determined by measuring its zeta potential, mean particle size, EE, and stability. The release rate of TP from SA/CMC/TP-Lip was determined to be pH controlled by simulating the release behaviour at different pH. The average release rate of TP encapsulated in TP-Lip was 41.08% at pH 3, which was 23.07% higher than that at pH 6 within 12 h. The mechanical and antioxidant properties of SA/CMC/TP-Lip were measured. The results showed that the addition of TP-Lip improved the tensile strength and antioxidant capacity of SA/CMC/TP-Lip by 30.55% and 7.16% respectively. From the application studies, the film effectively reduces the hardness, pH, and weight loss changes of blueberries, SA/CMC/TP-Lip can be used to maintain the freshness of blueberries during storage. Thus, novel films can be used to maintain fruit freshness during storage, and biodegradable controlled release films are expected to be an important packaging material in the future.

## Abbreviations

BBD: Box–Behnken design
CHOL: Cholesterol
CMC: Carboxymethylcellulose
Co.: Company
EAB: Elongation at break
EE: Encapsulation rate
FAO: Food and agriculture organization
FT: Freeze–thaw
FTIR: Fourier transform infrared spectroscopy
LOX: Lipoxygenase
Ltd.: Limited
PC: Lecithin high potency
RH: Relative humidity
ROS: Reactive oxygen species
SA: Sodium alginate
SEM: Scanning electron microscope
TP: Tea polyphenols
TP-Lip: Liposomes encapsulating tea polyphenols
TS: Tensile strength
TSS: Total soluble solid
WVP: Water vapor permeability

## Author contributions

Conceptualization, Xin Feng and Yang Li.; methodology, Xin Feng and Zhuoyu Cui.; software, Xin Feng and Rongrong Tang.; validation, Yang Li and Zhuoyu Cui.; data curation, Xin Feng and Zhuoyu Cui.; writing—original draft preparation, Xin Feng.; writing—review & editing, Xin Feng, Rongrog Tang and Yang Li.; supervision, Yang Li.; funding acquisition, Yang Li.

## Conflicts of interest

There are no conflicts to declare.

## Supplementary Material
